# ERPs Reveal Disengagement Processes Related to Condom Use Embarrassment in Intention-Behavior Inconsistent Young Adults

**DOI:** 10.1007/s10508-018-1217-4

**Published:** 2018-04-25

**Authors:** Phil Brüll, Loes T. E. Kessels, Linda Repetto, Anne Dirkson, Robert A. C. Ruiter

**Affiliations:** 1 Department of Work and Social Psychology, Maastricht University, 6200 MD Maastricht, The Netherlands; 2 University College Maastricht, Maastricht University, Maastricht, The Netherlands

**Keywords:** ERP, P300, Intention-behavior gap, Sexual health, Condom use, Embarrassment

## Abstract

The use of barrier protections such as condoms has consistently been reported to reduce the acquisition of sexually transmitted infections. However, it has also been reported that the association between condom use intentions and behavior is, at best, often weak. Furthermore, embarrassment associated with purchasing condoms and negotiating their use has been shown to negatively impact the frequency of condom use. Using electroencephalography to analyze P300 event-related potential components known to measure early attention allocation, we examined electrophysiological evidence of early attention disengagement for embarrassing health information. Forty young adults—34 females and six males—participated in an adapted version of Posner’s visual cueing paradigm. All were high in intention to use condoms, but half were intention-behavior consistent and half were intention-behavior inconsistent. Compared to intention-behavior consistent participants, those with intention-behavior inconsistency showed a reduced P300 component when attending to a visual target opposite to the field in which embarrassing self-relevant health information was presented, indicating more efficient early attention disengagement from such embarrassing health information. In conclusion, our electrophysiological data suggest that high intention alone may be not sufficient to predict adolescents’ condom use behavior.

## Introduction

The use of barrier protections such as condoms has been consistently shown to reduce the acquisition of STIs (e.g., Weller & Davis, 2002). Furthermore, a relationship between the intention to use condoms and actual condom use behavior has been demonstrated in multiple studies (see the meta-analysis by Albarracín, Johnson, Fishbein, & Muellerleile, 2001). Nevertheless, it has also repeatedly been reported that the association between condom use intention and actual behavior is often weak (Bauman, Karasz, & Hamilton, 2007; Fridlund, Stenqvist, & Nordvik, 2014; Sheeran & Orbell, 1998; Smith & de Visser, 2004; Turchik & Gidycz, 2012). In fact, behavioral intention appears to account for just 30% of the variance in actual behavior (Armitage & Conner, 2001; Sheeran, 2002). This inconsistency between intention and behavior (e.g., when people with positive intentions to perform a behavior fail to act on their intentions) is referred to as “the intention-behavior gap” (Sheeran, 2002). Given that intention-behavior gaps have been observed in relation to various different health-promoting behaviors, including physical activity, weight loss, and illicit drug use (Sheeran, 2002; Webb & Sheeran, 2006), it seems important to understand the mechanisms that determine the strength of the link between intention and behavior.

Assertive condom negotiation strategies (e.g., withholding sex and direct request for condom use) have been identified as the most important factors associated with increased condom use in both women and men (French & Holland, 2011). The use of these strategies can increase perceived self-efficacy in terms of condom negotiations; this, together with a low level of fear regarding the negotiation of condom use, and good communication with sex partners about condom use, has been shown to predict consistent condom use (Crosby et al., 2013). However, it has also been reported that the embarrassment associated with purchasing condoms—and negotiating their use—negatively impacts the frequency of condom use (Moore et al., 2008; Moore, Dahl, Gorn, & Weinberg, 2006). Embarrassment can be defined as a distinct emotion which threatens an individual’s social identity during an interaction (Keltner & Buswell, 1997). Furthermore, embarrassment has been linked to the belief that others might detect a personal flaw (Sabini, Garvey, & Hall, 2016), and has recently been shown to predict avoidance of sexual health sustaining behavior such as genital examinations and discussions of sexuality with health care professionals (McCambridge & Consedine, 2014). It is known that health-related information that is perceived to be self-relevant, but for which the recommended health action is perceived to be difficult to implement, is likely to induce defensive reactions (Peters, Ruiter, & Kok, 2012). This can be explained by Festinger’s (1957) dissonance theory and Kunda’s (1990) motivated reasoning approach. To date, defensive reactions related to embarrassment have not been studied extensively (Consedine, Krivoshekova, & Harris, 2007). Studies investigating the amount of attention that is allocated to health information (e.g., Brown & Locker, 2009; Liberman & Chaiken, 1992; Mogg, Bradley, De Bono, & Painter, 1997) have found that individuals who are most at risk react defensively to intimidating health messages by avoiding those messages or attending away from them. Discussing condom use with a new sex partner clearly entails a potentially embarrassing situation (Moore et al., 2006), which may, via avoidance, result in weakening—or even a disruption—of the intention-behavior link.

Electroencephalography (EEG) is a continuous recording of brain activity with Event-Related Potentials (ERPs) as time-locked measures of averaged cortical electrical activity representing a distinct phase of cortical processing. The P300 ERPs have a maximum positive peak around 300 ms after stimulus onset (Bentin, Mouchetant-Rostaing, Giard, Echallier, & Pernier, 1999) and are shorter over frontal areas and longer over parietal areas (Conroy & Polich, 2007; Polich, 1997). They show larger amplitudes when a target stimulus is attended to as compared to when little or no attention is allocated to the target stimulus (Kok, 1997). The P300 ERP component is therefore suitable for detecting early attention allocation (Böcker, Baas, Leon Kenemans, & Verbaten, 2004; Johnson, 1993; Polich, 2007), especially for self-relevant stimuli (Gray, Ambady, Lowenthal, & Deldin, 2004; Patel & Azzam, 2005). As shown by Kessels, Ruiter, and Jansma (2010), EEG can be used to investigate defensive responses to (threatening) health messages. They demonstrated that smokers for whom the information was self-relevant disengaged more efficiently (and thus used fewer attentional resources) from high-threat smoking pictures than from low-threat smoking pictures on invalid trials (where the target stimulus was presented on the opposite side of the screen as the cue). This was reflected in lower P300 amplitudes for smokers for high- as opposed to low-threat trials. For non-smokers, for whom the information was not self-relevant, no significant difference in P300 activity was found. Reduced P300 amplitudes have also been shown in women with hypoactive sexual desire disorder during the viewing of low-intensity sexual films as compared to high-intensity sexual films (Vardi et al., 2006). Prause, Steele, Staley, and Sabatinelli (2015) investigated the late positive potential (LPP), an ERP with an early time window of 300-600 ms reflecting facilitated attention to emotional stimuli (Schupp et al., 2000; Schupp, Junghöfer, Weike, & Hamm, 2004). They found that the LPP was reduced in response to less explicit sexual images for participants who reported fewer intercourse partners in the last year as compared to those who reported more sexual intercourse partners (and who showed similar LPP amplitudes to both more and less explicit sexual images). Furthermore, Krug, Plihal, Fehm, and Born (2000) reported an influence of the menstrual cycle on LPPs. This was demonstrated by an increased amplitude to sexual stimuli during the ovulatory phase, suggesting a greater valence of sexual stimuli during a phase that is marked by increased sexual desire.

In the present study, we examined whether negotiation regarding condom use induces an early attention disengagement process related to embarrassment. We expected that pictures depicting condom negotiation would induce embarrassment. Furthermore, we predicted that individuals with high intention to use condoms but with inconsistent (past) condom use behavior (intention-behavior inconsistent individuals), as compared to those with high intention to use condoms and consistent (past) condom use behavior (intention-behavior consistent individuals), would attempt to reduce their feeling of dissonance by disengaging attention from those pictures. Adopting the experimental paradigm used by Kessels et al. (2010), we therefore expected that intention-behavior inconsistent participants would make use of fewer attentional resources to detect visual targets presented in opposite areas of the visual field in which the uncomfortable cue was presented (invalid trials), as compared to individuals for whom the information was less self-relevant (i.e., participants with high intention and consistent (past) condom use behavior). In this attention cueing paradigm, reactions to targets in the invalid trials thus reflect disengagement processes.

As the use of attentional resources is reflected in both the amplitude of the P300 ERP and in reaction times as measured by a specific key press following stimulus presentation (e.g., Ito, Larsen, Smith, & Cacioppo, 1998), we expected participants with intention-behavior inconsistency to show lower P300 amplitudes and faster reaction times in invalid and embarrassing trials than intention-behavior consistent participants. Targets in valid trials (where the target stimulus is presented on the same side of the screen as the cue) were expected to induce similar patterns of results for behavior consistent and behavior inconsistent participants because these trials reflect mere attention-capturing processes.

## Method

### Participants

In total, 40 young adults (34 females, 6 males) aged between 18 and 25 years (*M *= 21 years, SD = 1.4) were selected for participation in the EEG study. Beforehand, a total of 245 undergraduate university students were recruited from various public places at the university as well as from social media sites. They completed a short online questionnaire that asked about demographics (age, sex), sexual identity (heterosexual, homosexual, bisexual, undecided), and current relationship status (in a romantic relationship, not in a romantic relationship). Next, their intention to use condoms during first-time sex with a new sex partner was assessed (“I intend to always use condoms when I have sex with a new sex partner for the first time”, using a 7-point Likert-scale, ranging from [1] “*unlikely*” to [7] “*likely*”). Finally, past condom use behavior was assessed (“Last time I had sex with a new sex partner for the first time, I used condoms”, using a 7-point Likert-scale, ranging from [1] “*false*” to [7] “*true*”). Scores on items that assessed the same variable were averaged into one measure in cases where internal consistency was sufficient (Cronbach’s alpha ≥ 0.70 with two items). Only participants who reported a high intention (6 or 7 on the 7-point Likert-scale) to use condoms during first-time intercourse with a new partner were selected. Within this high intention group, those respondents who reported having used a condom the last time they had intercourse with a new sex partner (6 or 7 on the 7-point Likert-scale) were asked to participate in the study. The first 20 respondents who agreed to participate formed the intention-behavior consistent group. Similarly, the first 20 respondents from the high intention group who reported not to have used a condom during their last first-time intercourse with a new sex partner (1 or 2 on the 7-point Likert-scale) comprised the intention-behavior inconsistent group. All participants gave their written informed consent as required by the Institutional Review Board and received a gift voucher of €25 after their participation. All participants were right-handed, had normal or corrected-to-normal vision, were heterosexual, and were sexually active but not involved in a romantic relationship at the time of the experiment.

## Materials

**Fig. 1:**
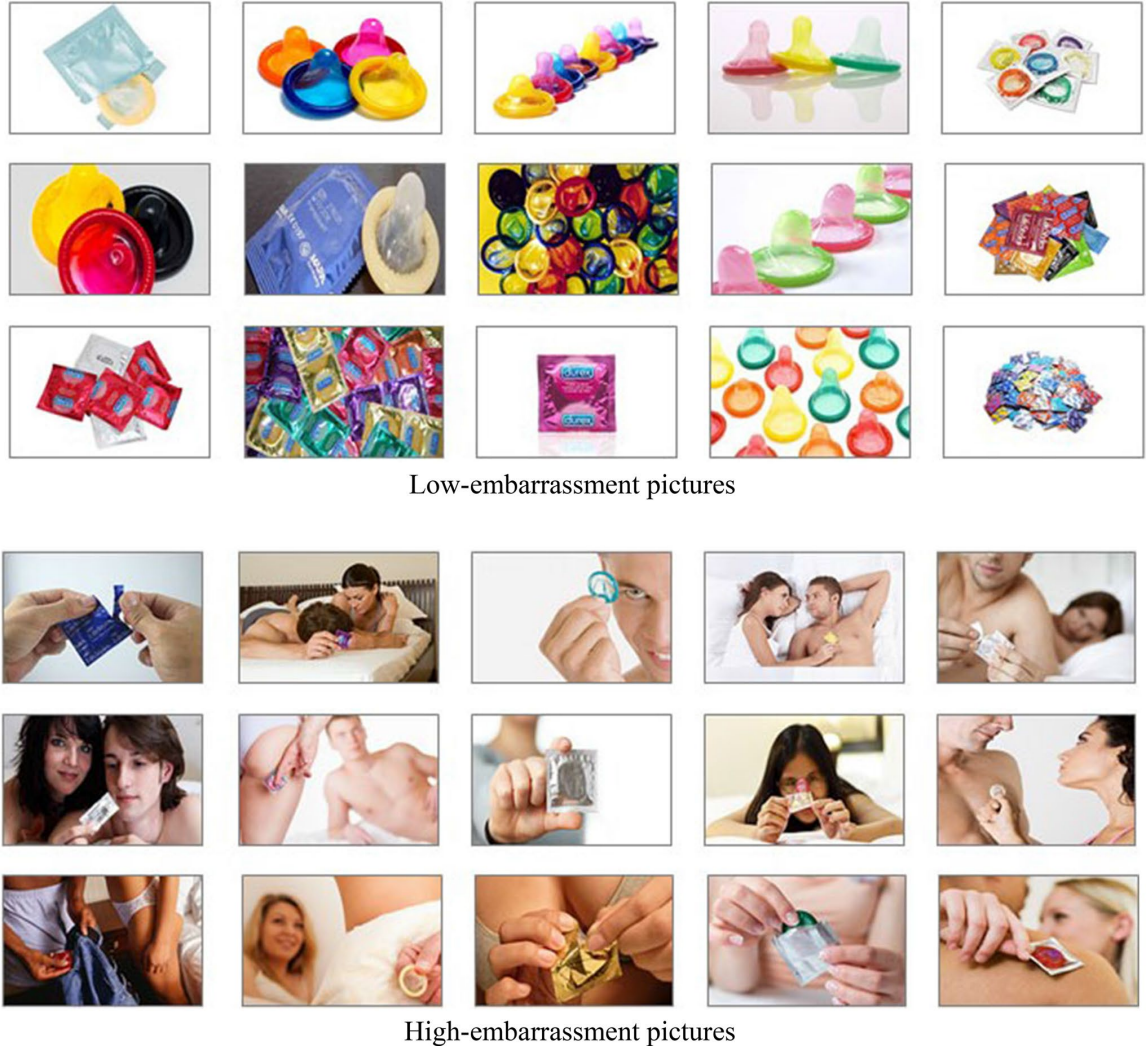
Fifteen color low-embarrassment pictures showing condoms and 15 high-embarrassment color pictures showing condoms that were used as cues in the cueing task

In total, 30 color pictures of condoms—either high or low in embarrassment—were used as cues in the cueing task. The low-embarrassment pictures showed only condoms while high-embarrassment pictures showed individuals holding a condom in their hands (see Fig. 1). The selection of these pictures was based on a pilot study in which 100 condom-related pictures were presented to 50 undergraduate students. For each picture, they were asked to rate their perceived level of discomfort on a 7-point Likert-scale (ranging from [1] “*not at all uncomfortable”* to [7] “*extremely uncomfortable”*) and embarrassment (ranging from [1] “*not at all embarrassed”* to [7] “*extremely embarrassed”*), Cronbach’s alpha = 0.71. Finally, 15 highly embarrassing (high-embarrassment) pictures with a minimum score of five and 15 only slightly embarrassing (low-embarrassment) pictures with a maximum score of three were selected and used as stimulus material. During the EEG experiment, the level of embarrassment level (high vs. low) and the validity of the trials (valid vs. invalid) were varied as within-subjects factors. Behavioral consistency in relation to behavioral intention (intention-behavior consistent vs. intention-behavior inconsistent) was included as a between-subjects factor.

### Procedure

**Fig. 2:**
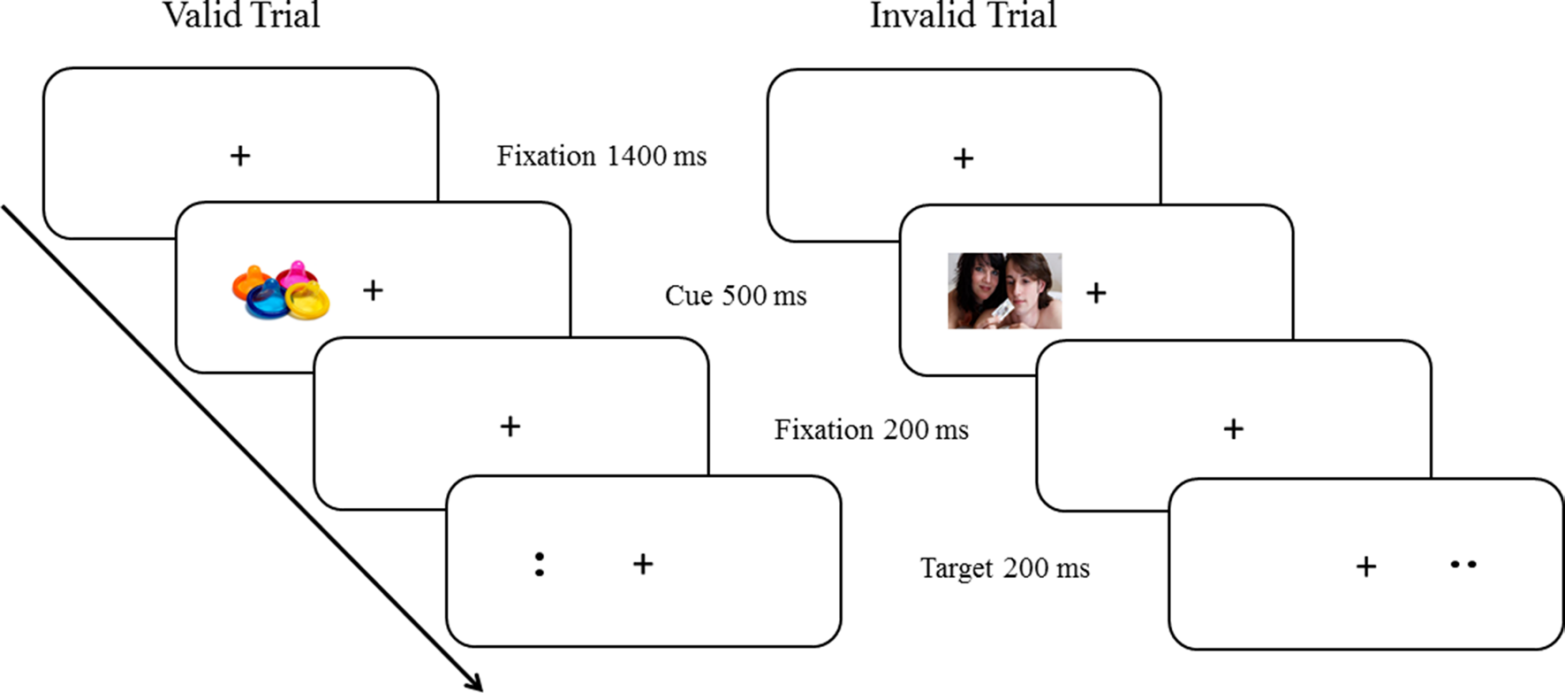
Experimental procedure showing an example of low-embarrassment pictures (valid trial) and high-embarrassment pictures (invalid trials)

For the cueing paradigm, pictures (255 × 170 pixels) were presented at the left or right side of the midpoint of a 17-inch computer screen with a distance of 8 cm. Two horizontal dots (··) or two vertical dots (:) were used as target stimuli. In each trial, a fixation cross was presented for 1,400 ms in the middle of the computer screen, followed by a high-embarrassment or a low-embarrassment cue for 500 ms (see Fig. 2). After a second fixation period of 200 ms, a target stimulus was presented for another 200 ms, either on the same side of the computer screen as the cue (valid trial: 82% of trials) or on the opposite side of the computer screen (invalid trial: 18% of trials). There were 1320 trials in total (cue and target), comprising 1080 valid trials and 240 invalid trials. Cues in the valid condition correctly indicated the position of the upcoming target whereas cues in the invalid condition appeared on the side opposite to the subsequent target. These cue-target combinations were randomly presented within each of the 33 experimental runs. The inter-trial interval was 2300 ms. After each block of 40 trials, participants were allowed to take a short break.

Participants sat approximately 80 cm from a computer screen on which the experimental stimuli were displayed. Before the start of the experiment, a priming screen with all of the high-embarrassment pictures was shown to the participants. They were asked to look at these pictures for 1 min and to imagine themselves being in that very situation, meaning a situation in which they had to negotiate condom use or take the initiative to use condoms. They were then once more instructed to imagine themselves being in such a situation for the time in which the pictures were presented on the screen. Participants were further instructed to keep their eyes fixated on the fixation cross and to press the left button as quickly as possible when two horizontal dots were presented and to press the right button as quickly as possible when two vertical dots were presented. They were also told that the probability of a valid trial was larger than the probability of an invalid trial. A practice block lasting 2 min allowed participants to familiarize themselves with keeping their gaze fixed on the fixation cross while responding correctly to the targets.

After the cueing task, participants were asked to evaluate the perceived level of embarrassment related to each picture on a 7-point Likert-scale (ranging from [1] “*not at all embarrassing”* to [7] “*extremely embarrassing”*). The entire experiment lasted approximately 2 h.

### Measures

#### Reaction times and errors

Button-press responses were measured from target onset with errors consisting of mistakes (e.g., the participant pressed right for horizontal dots and left for vertical dots), misses (no button press), and reaction times faster than 120 ms and slower than 1000 ms.

#### EEG and ERPs

The EEG was recorded from 21 scalp sites with Ag/AgCl-sintered electrodes, following the 10/20 system. Electrodes were mounted in an electrode cap, and the EEG recordings were referenced online to the left mastoid and re-referenced offline to the average of the left and right mastoids by subtracting one-half the amplitude of the right mastoid to the left mastoid recording. Horizontal eye movements were recorded with a bipolar montage of electrodes, placed on the right and left external canthus, and vertical eye movements and eye blinks were recorded on the upper and lower orbital ridge of the left eye. The electro-oculogram allowed that trials contaminated with eye movements were later rejected offline. All electrode impedances were kept below 5 kΩ, and signals were digitized at a 250 Hz sampling rate using a 32 channel BrainVision (Brain Products, Munich, Germany) amplifier. A band-pass filter of 0.05 to 30 Hz was used to filter the continuous EEG offline. From the continuous EEG signal, epochs of 1500 ms were obtained for ERP analysis, starting at 100 ms pre-cue onset (serving as a baseline) and ending 600 ms after target offset. Epochs containing artifacts beyond − 100 and 100 μV were removed before analysis. In total, 19% of the analyzed trials as well as the entire data sets of four participants could not be used due to excessive movement artifacts. This left a total number of 18 participants per condition: 16 females and two males aged between 18 and 24 years (*M* = 20 years, SD = 1.5) in the intention-behavior consistent group, and 15 females and three males aged between 18 and 23 years (*M* = 20 years, SD = 1.6) in the intention-behavior inconsistent group. There was no significant age difference between both groups, *t*(18) = 0.45, *p* = .31. The EEG sequences for each combination of embarrassment level, validity, and electrode site for each participant were averaged to ERPs. Finally, a grand average of these individual ERP data was calculated per experimental condition and electrode site for both intention-behavior consistent and inconsistent groups.

### Statistical Analyses

Mixed analyses of variance (ANOVAs) with embarrassment level (high vs. low) and validity (valid vs. invalid) as the within-subject factors and intention-behavior consistency (consistent vs. inconsistent) as the between-subjects factor were used to analyze reaction times and errors. ERP analyses were restricted to midline electrodes Fz, FCz, Cz, CPz, and Pz because attention effects are generally largest for these midline electrodes (Johnson, 1993; Polich, 2007). To control for an unequal distribution of valid and invalid trials, separate embarrassment level (high vs. low) × electrode (Fz, FCz, Cz, CPz, Pz) × behavioral consistency (consistent vs. inconsistent) mixed measures ANOVAs were performed for reactions to the valid and the invalid targets, respectively. To control for sphericity violations in the ANOVAs, probability values with Greenhouse–Geisser correction for *F* tests with more than 1 degree of freedom in the numerator are reported, and the reported estimates of the effect size are the partial eta squared (*η*_p_^2^) for the analyses of variance.

## Results

### Error Analysis

On average, more late (*M* = 29.19, SD = 40.68) than premature (*M* = 5.36, SD = 9.76) responses per participant were excluded, *t*(35) = 4.30, *p* < .001. As shown in Table 1, compared to intention-behavior consistent participants, intention-behavior inconsistent participants tended to make more errors across conditions, but these differences failed to reach statistical significance. No main effects or interactions involving the factors embarrassment, validity, and behavioral consistency were found (*p*’s > .175).

**Table 1:** Behavioral performance measures as a function of trial validity, embarrassment, and past behavior consistency

Variable	Valid				Invalid			
	High emb.		Low emb.		High emb.		Low emb.	
	M	SD	M	SD	M	SD	M	SD
Reaction time (ms)								
I-B consistent	540	72.21	549	75.69	564	74.43	571	77.83
I-B inconsistent	544	61.96	544	59.40	562	57.3	556	62.42
Errors (%)								
I-B consistent	6.22	28.87	5.74	24.57	7.06	6.08	6.62	5.05
I-B inconsistent	7.68	22.84	7.21	22.52	8.68	27.06	9.03	29.88

### Reaction Times

Mean reaction times (in ms) to the target stimuli (horizontal and vertical dots) are also shown in Table 1. As expected, reaction times to valid targets were faster (*M *= 540.34, SD = 48.52) than reaction times to invalid targets (*M *= 561.15, SD = 46.19), *F*(1, 17) = 47.96, *p* < .001. In addition, a significant main effect for embarrassment showed that reaction times to high-embarrassment targets (*M *= 548.15, SD = 46.69) were faster than to low-embarrassment targets (*M *= 553.34, SD = 47.55), *F*(1, 17) = 6.97, *p* < .005. No further main effects or interaction effects were found, *F’*s < 2.33, *p*’s > .80.

### Self-Report Measures

Intention-behavior inconsistent participants rated the embarrassment level of the high-embarrassment pictures significantly higher (*M* = 1.87, SD = 0.71) than the embarrassment level of low-embarrassment pictures (*M* = 1.45, S*D* = 0.43), *t*(17) = 2.85, *p* < .05, *d* = .71. Furthermore, intention-behavior consistent participants also rated the level of embarrassment significantly higher for high-embarrassment (*M* = 1.83, SD = 0.77) versus low-embarrassment (*M* = 1.35, SD = 0.44) pictures, *t*(17) = 3.36, *p* < .05, *d* = .76. However, there was no significant difference between intention-behavior consistent and intention-behavior inconsistent participants for the ratings of high-embarrassment pictures, *F*(1, 35) = 0.32, *p* = .85, and of low-embarrassment pictures, *F*(1, 35) = 0.53, *p* = .47.

### ERP Analyses

#### Visual Inspection

**Fig. 3:**
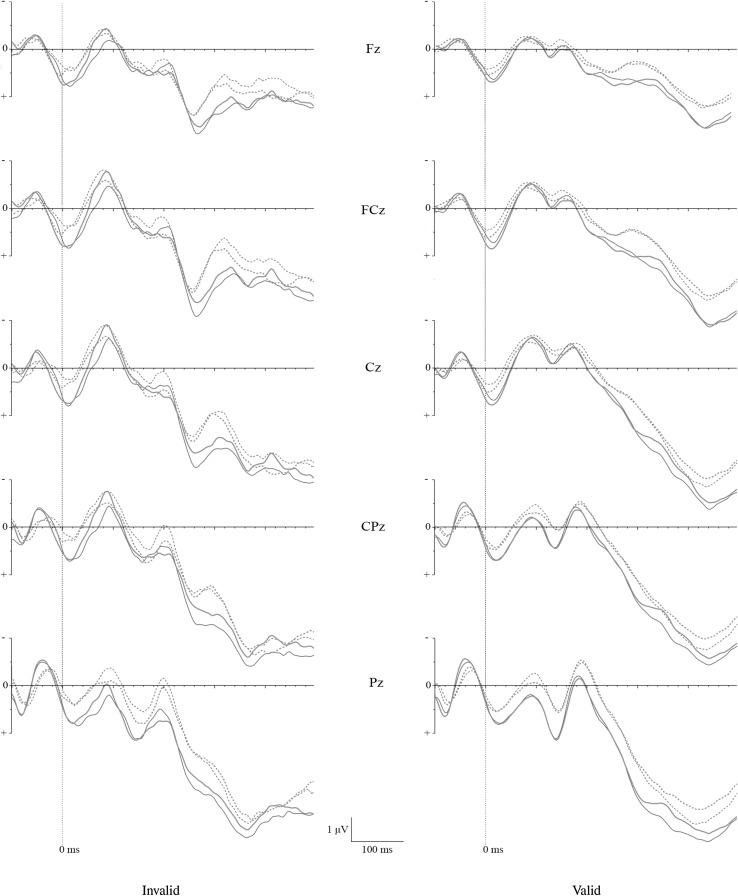
Grand average P300 event-related potentials (ERPs) for high- and low-embarrassment stimuli across intention-behavior consistent and inconsistent groups in invalid and valid trials: high embarrassment, intention-behavior consistency (thin full line); high embarrassment, intention-behavior inconsistency (thin dashed line); low embarrassment, intention-behavior consistency (thick full line); low embarrassment, intention-behavior inconsistency (thick dashed line)

Visual inspection of the ERP waves in reaction to the target stimuli showed the expected P300 effect on the midline electrodes Fz, FCz, Cz, CPz, and Pz (Table 2). The grand average ERP waveforms indicate that the expected P300 effect for the invalid trials was most strongly concentrated at the frontal-central electrodes between 240 and 340 ms after target onset (Fig. 3). Based on this visual inspection, we concentrated our separate analyses on the Fz, FCz, and Cz electrodes.

**Table 2:** Amplitudes (μV) of the P300 effect as a function of trial validity, level of embarrassment, and behavioral consistency

Electrode	Valid				Invalid			
	High emb.		Low emb.		High emb.		Low emb.	
	M	SD	M	SD	M	SD	M	SD
Fz								
Consistent	3.84	2.05	3.77	1.94	3.10	2.11	3.25	1.96
Inconsistent	3.05	2.20	3.08	2.05	2.04	2.52	1.86	2.22
FCz								
Consistent	4.93	2.49	5.28	2.43	5.65	1.90	4.75	2.38
Inconsistent	4.16	2.63	4.29	2.80	3.91	2.86	3.12	3.28
Cz								
Consistent	5.79	2.42	6.39	2.99	7.07	1.60	5.32	2.16
Inconsistent	5.16	3.04	5.57	3.10	5.01	3.45	3.51	3.41
CPz								
Consistent	6.83	2.43	7.36	2.22	7.08	1.69	7.79	2.14
Inconsistent	6.00	3.15	6.52	3.37	6.52	3.68	6.16	3.63
Pz								
Consistent	7.33	2.50	7.89	2.39	7.67	1.66	8.53	2.12
Inconsistent	6.09	2.93	6.74	3.27	6.73	3.47	6.68	3.48

#### P300

An overall mixed ANOVA for all five midline electrodes for the invalid trials showed a significant three-way interaction between electrodes, embarrassment, and intention-behavior consistency, *F*(4, 31) = 3.40, *p* < .05, *η*_p_^2^ = .27. There was a further significant interaction effect between embarrassment level and electrodes, *F*(4, 31) = 3.12, *p* < .05, *η*_p_^2^ = .28, and a significant main effect of electrodes, *F*(4, 31) = 10.76, *p* < .001, *η*_p_^2^ = .58.

More specific mixed ANOVAs for the invalid trials on the Fz, FCz, and Cz electrodes, where the P300 effect was most strongly concentrated, also showed a significant three-way interaction between embarrassment level, intention-behavior consistency and electrodes, *F*(2, 33) = 6.01, *p* < .05, *η*_p_^2^ = .26, a significant main effect of embarrassment level, *F*(1, 34) = 5.94, *p* < .05, *η*_p_^2^ = .14, and a significant main effect of electrodes, *F*(2, 33) = 60.58, *p* < .001, *η*_p_^2^ = .78. Mixed ANOVAs for the invalid trials on the CPz and Pz electrodes revealed only a main effect of electrodes, *F*(1, 34) = 7.21, *p* < .05, *η*_p_^2^ = .17.

Simple effect analyses for each level of embarrassment showed a significant interaction between intention-behavior consistency and electrodes for both high-embarrassment invalid trials, *F*(2, 33) = 10.13, *p* < .001, *η*_p_^2^ = .38, and for low-embarrassment invalid trials, *F*(2, 33) = 7.65, *p* < .001, *η*_p_^2^ = .75. In the high-embarrassment condition, P300 amplitudes at the three electrodes Fz, FCz, and Cz were significantly lower for intention-behavior inconsistent participants as compared to intention-behavior consistent participants, *F*’s>* 10.26*, *p*’s < .003, *η*_p_^2^’s > .23. These effects were largest at electrode Cz and smallest at electrode Fz. P300 amplitudes in the low-embarrassment condition at all three electrodes were also significantly lower for intention-behavior inconsistent participants as compared to intention-behavior consistent participants, *F*’s>* 13.06*, *p*’s < .001, *η*_p_^2^’s > .27.

Separate analysis for each level of intention-behavior consistency for invalid trials showed a significant interaction between embarrassment level and electrodes for both intention-behavior inconsistent participants, *F*(2, 16) = 4.96, *p* < .05, *η*_p_^2^ = .38, and for intention-behavior consistent participants, *F*(2, 16) = 7.61, *p* < .005, *η*_p_^2^ = .48. While intention-behavior inconsistent participants showed no significant difference in P300 amplitudes between high- and low-embarrassment trials across the three electrodes (*F*’s<* 3.33*, *p*’s > .86, *η*_p_^2^’s < .16), intention-behavior consistent participants showed significantly lower P300 amplitudes for low-embarrassment trials as compared to high-embarrassment trials across the three electrodes, *F*’s>* 17.54*, *p*’s < .001, *η*_p_^2^’s > .49. These effects were again largest at electrode Cz and smallest at electrode Fz.

The mixed ANOVA for all five midline electrodes for the valid trials did not show any interaction effects (*F*’s < .86, *p*’s > .22, *η*_p_^2^’s < .17) but revealed a significant main effect of electrodes *F*(4, 31) = 38.24, *p* < .001, *η*_p_^2^ = .69. Visual inspection of the grand average waveforms of the midline electrodes (Fig. 2) suggests larger amplitudes of the P300 effect for parietal electrodes as compared to frontal-central electrodes (see also Table 2).

## Discussion

Using a visual selective attention paradigm, we found support for our hypothesis that young adults with intention-behavior inconsistency (as related to condom use) displayed more efficient disengagement from both high- and low-embarrassment pictures displaying condoms and condom use negotiations than intention-behavior consistent participants. In fact, for invalid trials, intention-behavior inconsistent participants had lower P300 peak amplitudes for both low- and high-embarrassment trials as compared to intention-behavior consistent participants. Interestingly, compared to intention-behavior consistent participants, participants with past condom use-related intention-behavior inconsistency showed a significantly decreased P300 amplitude in invalid trials in response to condom-related pictures, independent of the level of embarrassment. In fact, compared to intention-behavior consistent participants, intention-behavior inconsistent participants disengaged attention more efficiently from even slightly embarrassing (low embarrassment) pictures. This could be related to feelings of self-threat, in line with Festinger (1957) and Kunda (1990) who proposed that people want to reduce feelings of cognitive dissonance by altering the respective cognitive or behavioral elements. Intention-behavior inconsistent participants may do this by already attending away from even slightly embarrassing information.

We did not, therefore, find support for our prediction that intention-behavior inconsistent young adults disengaged attention more efficiently from pictures they rated as highly embarrassing (pictures showing condom use negotiations) than from pictures they rated as slightly embarrassing (pictures showing condoms only). Rather, we found that intention-behavior consistent young adults disengaged attention more efficiently from low-embarrassment pictures than from high-embarrassment pictures, as indicated by lower P300 peaks for low-embarrassment stimuli compared to high-embarrassment stimuli in invalid trials. This finding may be in line with previous research that showed higher P300 amplitudes for self-relevant stimuli compared to neutral stimuli (e.g., Gray et al., 2004). One could, in fact, argue that for those individuals who act upon their health intentions, mere pictures of condoms are perceived as less self-relevant than pictures showing familiar condom negotiation situations, resulting in lower P300 amplitudes.

As indicated by our ERP results in invalid trials, attention disengagement can arise within 300 ms after stimulus presentation and reflects an early process of attention disengagement from embarrassing health information. Even if participants with inconsistent past condom use behavior explicitly reported their intention to use condoms during first-time sex with a new partner, it was the embarrassing information related to condoms that may have caused the observed involuntary shift of spatial attention, referred to as attentional capture by Remington, Folk, and McLean (2001). These participants attended to stimulus properties relevant to their implicit behavioral goals, namely to avoid a potentially embarrassing situation. Such an involuntary attention shift as shown in our ERP results has been proposed to be contingent on the relationship between the eliciting event and the required task performance (Folk, Remington, & Johnston, 1992). According to the central tenet of Edelmann’s (1985) embarrassment theory, protective self-presentation can conflict with knowledge of social rules. Our study revealed the persistence of embarrassment in a group of intention-behavior inconsistent young adults and underscores the importance of directing coping strategies at overcoming this embarrassment. In fact, simply having good intentions to use condoms may not result in actual condom use (the intention may not be translated into behavior) if condoms and condom use are associated with potentially embarrassing situations. It follows, then, that embarrassment associated with condom use negotiation could represent a barrier to consistent condom use with a new sex partner among young adult populations.

However, embarrassing and control images may have differed on other dimensions besides embarrassment level, namely on erotic content and inclusion of people. It is possible that the attentional disengagement could have been due to differing responses to the higher level of erotic content in the embarrassing images. Erotic stimuli have been shown to involuntarily draw one’s attention, just as visual orientation is drawn toward threat-related stimuli (Sennwald et al., 2016). This constitutes an alternative explanation for the higher P300 for high- versus low-embarrassment images within the intention-behavior consistent group. It may also explain the higher P300 and increased attentional engagement in the intention-behavior consistent group for the embarrassing images (if indeed they did find them more erotic than the intention-behavior inconsistent participants).

Alternatively, the inclusion of people only in the embarrassing images could have influenced attentional processing, as participants may respond differentially to social situations. For instance, individuals with social phobia and anxiety show difficulties with attentional disengagement from socially threatening social stimuli (Amir et al., 2010; Amir, Elias, Klumpp, & Przeworski, 2003; Bantin, Stevens, Gerlach, & Hermann, 2016; Beard, Sawyer, & Hofmann, 2012; Heeren, Mogoase, Philippot, & McNally, 2015). On the other hand, the majority of studies indicate that individuals with autism spectrum disorder have decreased visual attention to social stimuli compared to healthy individuals (Bellocchi, Henry, & Baghdadli, 2017). While we are not suggesting that our participants fulfill any of these clinical criteria, these characteristics do exist on a spectrum within the general population. The possibility that higher levels of social avoidance may also have contributed to the intention-behavior inconsistency via a reduced ability to communicate about condom use is also worth noting.

Attention disengagement can be decreased by increasing the proportion of targets appearing at the location of the intended training bias stimuli in a dot probe task (Lazarov & Bar-Haim, 2016). It has been shown that such attention bias modification training (ABMT) aimed at directing attention toward stimuli can significantly increase attentional bias toward and consumption of healthy food (Kakoschke, Kemps, & Tiggemann, 2014) and alcohol (Field & Eastwood, 2005) in healthy participants. Furthermore, ABMT has been shown to be effective in reducing negative—or threat-related—attention bias by directing attention away from threat-relevant stimuli in healthy controls facing a stressful situation (Mogoaşe, David, & Koster, 2014) as well as in various patient groups, such as adolescents with depression (Lazarov & Bar-Haim, 2016) or individuals with social phobia (Amir et al., 2010) or anxiety (Beard et al., 2012; Heeren et al., 2015).

Given these results, it may also be feasible to increase attention toward condom-related stimuli, thereby decreasing attention disengagement, using ABMT. However, such interventions remain challenging because internet-based ABMT on personal computers has not been shown to have any significant effect (Boettcher et al., 2013; Boettcher, Berger, & Renneberg, 2012; Carlbring et al., 2012; Neubauer et al., 2013). Alternatively, smartphones remain a potential medium, but more research is necessary. To our knowledge, only one study has used smartphones to deliver ABMT to individuals with social anxiety, resulting in a small effect on attention bias scores (Enock, Hofmann, & McNally, 2014).

Our prediction that intention-behavior inconsistent participants would show faster reaction times than intention-behavior consistent participants in response to high-embarrassment pictures was not confirmed. One possible reason for this could be the large response speed variability in our sample (Van Damme & Crombez, 2009). However, ERP waves showed significant differences between both groups in time windows that preceded response preparation, indicating that attention toward embarrassing health information is modulated at an early stage of cognitive processing. As expected, we found an effect of embarrassment across both groups as reflected in faster reaction times, and hence faster disengagement, in the highly embarrassing condition. Furthermore, an effect of validity was identified across both groups, indicating faster responses to valid as opposed to invalid trials. The latter finding is in line with Pollak and Tolley-Schell (2003), who showed faster reaction times in cueing tasks for valid as compared to invalid trials. However, unlike Pollak and Tolley-Schell, we did not find higher accuracy rates for valid over invalid trials. Indeed, in our study, intention-behavior consistent participants showed less accuracy in valid trials, independent of embarrassment, and no significant difference was found between valid and invalid trials for intention-behavior inconsistent participants. We can only speculate that intention-behavior consistent participants may have reduced their attention to the target due to the limited self-relevance of the previously ipsilateral presented cue, whereas contralateral presented targets may have increased participants’ attention due to their new position on the screen.

There are several possible limitations to our study. First, our sample consisted of 31 female and five male participants. While taking into account the finding that women’s perceived relationship power control has not been found to be associated with safer-sex practices (Hahm, Lee, Rough, & Strathdee, 2012), we nevertheless cannot exclude the possibility that a gender bias related to intention and past behavior measures influenced our results. Second, our study did not include a control group, so we cannot infer a causal link between embarrassment and condom use behavior. It is worth noting that we did not ask participants about their recent experiences with sex-related or condom-related negotiations, meaning that we cannot exclude the possibility that recent adverse experiences may have influenced our findings. Third, the cueing task consisted of low-embarrassment pictures that showed condoms only, while high-embarrassment pictures showed individuals holding a condom in their hands—thus inferring a situation involving condom use negotiation. Even though participants were asked to imagine themselves in that fictional situation—and thus imagine being involved in a negotiation about condom use—we cannot exclude the possibility of a response bias occurring related to the differing content (condoms versus condoms + individuals) of these pictures. Lastly, no-cue conditions are commonly used in behavioral studies using a cueing task. In a no-cue condition, target stimuli are presented without previous warning, allowing the investigation of an effect of validity on reaction times (e.g., Baayen & Milin, 2010; Perchet & García-Larrea, 2000). The present study did not use such no-cue conditions. While using ERP measures, no-cue trials could act as a confounding variable because of the added factor of surprise due to unanticipated stimuli (Jonides & Mack, 1984; Pollak & Tolley-Schell, 2003). The fact that we did not include no-cue conditions means that we cannot determine whether the effect of validity we found for reaction times reflects a cost related to an invalid cue or a benefit related to a valid cue.

In conclusion, the present study showed that, compared to intention-behavior consistent young adults, those with intention-behavior inconsistency disengaged attention more efficiently from low- and high-embarrassment pictures related to condoms and condom use. This was independent of their high intentions to use condoms during their next first-time sex with a new partner. Our findings suggest that a high level of intention alone may be insufficient to predict condom use behavior if its implementation is associated with feelings of embarrassment. Future research could address some of the limitations of this study. We recommend questioning subjects about the subjective eroticism of the images, in addition to assessing their general social skills. Another recommendation would be to utilize a more extensive set of control stimuli: condoms alone, people alone and condoms combined with people.
